# Editorial on the Special Issue on Recent Advances of Molecular Machines and Molecular Robots

**DOI:** 10.3390/mi11121031

**Published:** 2020-11-24

**Authors:** Masahiro Takinoue, Ryuji Kawano

**Affiliations:** 1 Department of Computer Science, Tokyo Institute of Technology (Tokyo Tech), 4259-J2-36 Nagatsuta-cho, Midori-ku, Yokohama, Kanagawa 226-8502, Japan; 2 Department of Biotechnology and Life Science, Tokyo University of Agriculture and Technology, Naka-cho 2-24-16, Koganei, Tokyo 184-8588, Japan

Molecular machines and molecular robots are a highly interdisciplinary research field including material science, chemistry, biotechnology, biophysics, soft matter physics, micro-electromechanical systems (MEMS), and computer science [1,2,3]. Molecular machine engineering is based on motor protein science and supramolecular chemistry and is currently expanded to the development of dynamical molecular machinery. Besides, the research field of molecular robotics originates from DNA nanotechnology and DNA computing and has recently yielded results in the construction of dynamical molecular machinery by taking advantage of the characteristics of a sequence-based programmable design. The interaction between these two fields will, furthermore, promote the development of nanometer- or micrometer-sized dynamical and programmable robotic systems equipped with molecular sensors and molecular intelligence.

In this Special Issue, six research papers and two review articles related to molecular machine engineering and molecular robotics from a wide range of research fields have been presented. Three theoretical research papers, on reaction–diffusion simulation accelerated the finite-element method by Sellami et al. [4], on a structural design of conjugated proton cranes based on the density functional theory (DFT) by Georgiev et al. [5], and on the modeling of mesh structure for a microtubule filament by Ueno et al. [6], will contribute the design for the molecular machines/robots. The other three research papers will be an insight into the construction or motion of molecular machines/robots. Liu et al. show a DNA ring motif that will be a useful tool for flexible joints [7], and Fujiwara et al. demonstrate honeycomb-patterned droplets with a lipid bilayer formed by a microfluidic device [8]. The self-reproducing artificial cell described by Matsuo et al. [9] establishes an environment-sensitive intelligence. Two review articles on liposome-based molecular robots, by Shoji et al. [10], and microfluidic artificial cell formation, by Kamiya [11], introduce the state of the art on the microfabrication/microfluidic-based construction for molecular robots.

By overviewing the recent advances in this field, we would like to ferment the seeds of future applications, such as medical microrobots, intelligent drug delivery systems, artificial cells/organelles, environmental nano/microsensor robots, agricultural nano/microrobots, and unconventional brain-like computers. We would like to thank all of the contributors to publish this Special Issue and we are happy if this issue helps to understand the recent advances in molecular machines and molecular robotics.
